# Is Being Male a Marker of Aggression? Evidence for the Decoupling of Sex and Gender Role Orientation

**DOI:** 10.3390/brainsci14121176

**Published:** 2024-11-25

**Authors:** Ziang Li, Yutong Liu, Weijun Liu, Hong Chen

**Affiliations:** 1 Faculty of Psychology, Southwest University, Chongqing 400715, China; liziang20061122@163.com (Z.L.); liuweijunpsy@163.com (W.L.); 2 Research Center of Psychology and Social Development, Southwest University, Chongqing 400715, China; 3 Key Laboratory of Cognition and Personality, Ministry of Education, Southwest University, Chongqing 400715, China; 4 Department of Applied Psychology, Harbin Normal University, Harbin 150500, China; liuyutong1214@163.com

**Keywords:** sex, gender role orientation, reactive aggression, proactive aggression, inferior frontal gyrus

## Abstract

Objectives: This study explores whether sex differences in reactive aggression (RA) and proactive aggression (PA) are attributable to sex, gender role orientation, or their interaction and explores the neuroanatomical characteristics of these sex differences. Methods: In a sample of 108 males and 126 females, we examined the sex-by-gender role orientation interaction on RA, PA, and brain gray matter volume (GMV). Then, we explored the relationship between aggression and regional GMV. Results: When the effects of sex and gender role orientation on aggression were disentangled, there were no sex differences in RA, regardless of gender role orientation. However, sex differences (male > female) in PA were observed within the masculine group but not within the feminine group. Brain imaging results revealed sex differences (male > female) on the right inferior frontal gyrus GMV, a region involved in cognitive control, within the masculine group. Moreover, a negative association between PA and the right inferior frontal gyrus GMV was observed in masculine females rather than masculine males. Conclusions: These findings indicate that gender role orientation has a more significant effect on aggression than sex, particularly with regard to PA, and hint that the goal of cognitive control involved in displaying PA differs in masculine males and masculine females.

## 1. Introduction

Aggression is one of the most sexually dimorphic behaviors [1], commonly categorized into two types: reactive aggression (RA) and proactive aggression (PA), which are distinguished by their underlying motivational factors [2]. RA is typically characterized by impulsivity and negative emotional states, such as anger [3], while PA is defined as deliberate, instrumental aggression with limited emotional reactivity [4], often associated with callous, unemotional traits [5]. Laboratory studies have shown sex differences in both RA and PA, with higher levels reported in boys [6], male adolescents [7], and adult men [8] compared to women. However, these results were interpreted within the framework of sex. In other words, previous studies have not clearly distinguished the differential effects of sex and gender role orientation on RA and PA.

Gender role orientation refers to the extent to which an individual identifies with personal attributes, characteristics, and personalities considered desirable for typical males or females in a given society [9]; these include masculinity traits (e.g., independence and assertiveness) and femininity traits (e.g., compassion and warmth) [10]. Individuals can be classified into masculine (masculinity ≥ median scores, femininity < median scores) or feminine (masculinity < median scores, femininity ≥ median scores) groups based on median scores for masculinity and femininity [11] or other methods. Prior research has linked masculinity/femininity to aggression. For example, males more likely to perpetrate sexual violence often exhibit high levels of masculinity [12]. Furthermore, males who display pronounced masculinity demonstrate higher aggression levels and report more assaults against females [13]. Similarly, a positive correlation has also been found between masculinity and RA/PA [14]. In contrast, femininity emphasizes much lower aggression [15]. For instance, femininity correlates negatively with self-reported aggression in females [16]. Additional studies support the inverse relationship between femininity and various types of aggression, including verbal [17], direct [18], and both RA and PA [14]. These results were observed in either males or females without accounting for sex as a factor. In general, individuals exhibiting masculinity traits are more likely to display high levels of RA and PA compared to those exhibiting femininity traits [19,20].

As previously stated, both males and masculine individuals tend to be more prone to high levels of RA and PA, while females and feminine individuals exhibit the opposite pattern. Sociocultural factors appear to cause the development of more masculine traits in males and more feminine traits in females [21]. This male–masculine/female–feminine congruency in children and adolescents’ developmental trajectories is strongly influenced by gender role expectations from parents, peers, and society during socialization [20]. Consequently, the effects of sex and gender role orientation on aggression remain unclear, as they have not been fully distinguished. It is possible that behind the sex differences in aggression found in the current literature are the effects of gender role orientation on aggression. Thus, it is essential to examine the interaction between sex and gender role orientation on aggression to better understand the factors that contribute to the sex differences in RA and PA.

Furthermore, examining the neural characteristics associated with gender role orientation in the two subtypes of aggression could enhance our understanding of the mechanisms underlying aggression and guide the development of future prevention programs or intervention strategies. Prior studies have revealed correlations between gray matter volume (GMV) in the straight gyrus and femininity in adults and girls [22,23]. Additionally, a positive correlation has been found between masculinity and white matter volume in the frontal lobe, as well as between femininity and gray matter volume (GMV) in the temporal lobe, among participants aged 7 to 17 [24]. Moreover, femininity correlates with GMV in males’ left middle frontal gyrus but not in females [25]. Overall, these structural magnetic resonance imaging (MRI) findings suggest that both sex and gender role orientation impact regional cortical volumes, particularly in the frontal lobe [26].

More importantly, RA and PA are closely linked to extensive frontal regions, particularly the prefrontal lobe [27]. A study demonstrated that increased RA levels are associated with reduced middle frontal cortex volumes [28]. Gallucci found that experimentally induced frustration increased RA following transcranial direct current stimulation (tDCS) of the left ventrolateral prefrontal cortex [29]. Participants who received anodal tDCS on the right dorsolateral prefrontal cortex experienced reduced PA [30]. Similarly, research has shown a negative association between PA and lateral and medial frontal cortex volumes in both normative and clinical populations [28,31,32]. Overall, these results suggest that the frontal regions, particularly the prefrontal cortex, play a crucial role in both RA (linked to impulsivity and negative emotions) [33] and PA (linked to low emotionality and cognitive control) [32].

Based on the literature discussed and the roles of masculinity and femininity in increasing or inhibiting aggression, we first hypothesized that sex differences in aggression would widen in the masculine group and narrow in the feminine group. Second, we hypothesized that the sex-by-gender role orientation interaction would influence frontal lobe volume. These regions may be closely linked to RA and PA. Therefore, we finally explored potential associations between GMV in significant regions and the two types of aggressive behaviors.

## 2. Methods

### 2.1. Participants

The data for this study were extracted from the Behavioral Brain Research Project on Chinese Personality [34], which initially included 906 participants. All participants were recruited through online advertisements and notices posted at local universities. After excluding those with incomplete data, 705 participants remained. Subsequently, these 705 participants were categorized into two groups—masculine (both masculinity ≥ 4.50 and femininity < 4.72) and feminine (both masculinity < 4.50 and femininity ≥ 4.72)—based on their median scores on the masculinity and femininity traits, as assessed by the Bem Sex Role Inventory [9]. Following this categorization, a total of 234 participants (108 males and 126 females, age range = 17.07–22.42, *M*_age_ = 19.10 ± 0.90) were selected for the current study. Of the total male participants, 69 (63%) were categorized as masculine and 39 (37%) as feminine. Similarly, of the total female participants, 42 (33%) were classified as masculine and 84 (67%) as feminine (Table 1). Within the masculine group, masculinity was significantly higher than femininity, while within the feminine group, femininity was significantly higher than masculinity, regardless of sex. It is worth noting that there was no significant difference between sexes in terms of femininity and masculinity, except that feminine females scored higher on femininity than feminine males (Table 2).

### 2.2. Procedure and Measures

The University Ethics Committee for Scientific Research reviewed and approved this study to ensure compliance with established ethical standards for treating human participants. All participants provided written informed consent. Consent was obtained from parents or guardians for subjects who were minors. Participants then underwent an MRI, during which they were instructed to keep their heads still and awake. Finally, all participants completed the required experiment questionnaires and received monetary compensation at the end of the study [35].

We measured sex, gender role orientation, RA, and PA. To comply with the text length limit, please refer to Part A of Supplementary Material S1.

### 2.3. MRI Data Acquisition and Processing

Structural scanning was conducted on a 3.0-T Siemens Trio MRI scanner (Siemens Medical, Erlangen, Germany). MRI images were obtained using a magnetization-prepared rapid gradient-echo T1-weighted sequence. All parameters were set as follows [36]: repetition time (TR) = 2530 ms; echo time (TE) = 2.98 ms; inversion time (TI) = 900 ms; flip angle = 7°; resolution matrix = 256 × 256. We acquired 176 contiguous sagittal slices with 1.0 mm slab thickness for the whole brain. The voxel size was 0.5 × 0.5 × 1 mm^3^. The scanning procedure followed the guidelines of the Research Project Ethical Review Committee.

The structural MRI images were processed using the SPM12 (Statistical Parametric Mapping, Welcome Department of Imaging Neuroscience, http://www.fil.ion.UCL.ac.UK/spm/, accessed on 23 November 2023). Following the procedures of previous studies [37,38]. More details are provided in Part B of Supplementary Material S1.

### 2.4. Statistical Analysis

The data for all behaviors were analyzed using SPSS (Version 24.0). The sample characteristics were analyzed using descriptive statistics in the preliminary analyses. The relations between the study variables were examined using Person correlation analyses. Then, we performed a two-way multivariate analysis of variance (MANOVA) on aggression and brain GMV, with sex and gender role orientation as factors. In accordance with the a priori objectives of this study (i.e., sex difference in aggression in different gender role orientations), we will perform simple effect analyses (i.e., pairwise comparisons) regardless of whether interactions are significant or not.

For brain structural data, a voxel-wise analysis of the interaction between sex and gender role orientation was performed using a full factorial design for brain structural data in SPM12. In the analysis, age and total intracranial volume (TIV) were entered as nuisance covariates. Meanwhile, an absolute threshold masking of 0.2 was applied to these analyses to exclude edge effects between white and gray matter [39]. The displayed and corrected results were completed using the DPABI software toolbox (Version 6.0) [40] in the MATLAB platform (Version 2018). The results were corrected using the Gaussian random field (GRF) program for multiple comparisons (threshold: cluster *p* < 0.05 and voxel level *p* < 0.001) [41,42]. Then, similar to the analyses of behavioral data, significant regions with interaction effects and main effects were selected as regions of interest (ROIs) for analysis using SPSS. The REX toolbox (https://www.nitrc.org/frs/?group_id=56; accessed on 28 November 2023) was used to obtain GMV means. Last, a Person correlation analysis was conducted on GMV in significant brain regions and the two aggressive behaviors.

## 3. Results

### 3.1. Descriptive Statistics and Correlation Analyses

**Figure 1:**
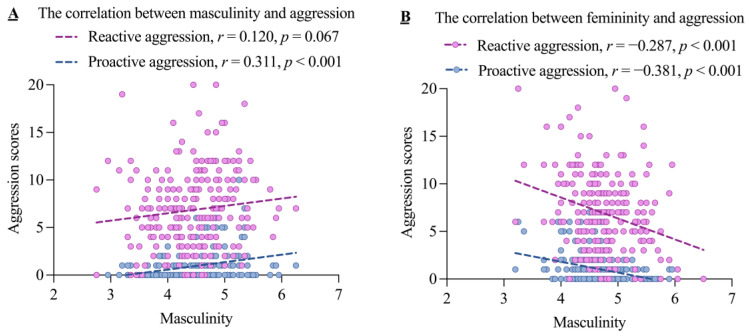
The correlations between masculinity/femininity and the two subtypes of aggression.

Table 3 displays the means and standard deviations for RA and PA across the four groups. Figure 1 visualizes the correlations between masculinity/femininity and RA/PA. Masculinity was marginally positively related to RA and positively related to PA, while femininity was negatively related to both RA and PA.

### 3.2. Sex Differences in RA Across Different Gender Role Orientations

**Figure 2:**
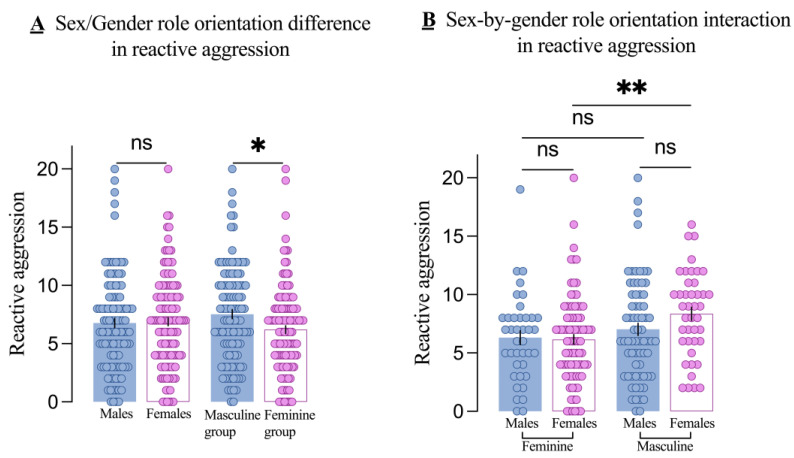
Main effects and interactions on reactive aggression. The main effect of sex (males and females) and gender role orientation (masculine and feminine groups) in reactive aggression (**A**) and the sex-by-gender role orientation interaction on reactive aggression (**B**). Each dot is a participant. All error bars reflect the standard error of the mean. ns = not significant. * *p* < 0.5; ** *p* < 0.001.

There was no significant sex-by-gender role orientation interaction on overall aggression in the two-way MANOVA (Wilks’ Λ = 0.98, F_2,229_ = 2.49, *p* = 0.085, partial η^2^ = 0.021). Regarding RA, as shown in Figure 2A, there was no significant main effect of sex (F_1,230_ = 1.17, *p* = 0.280, partial η^2^ = 0.005), but there was a significant main effect of gender role orientation (F_1,230_ = 7.24, *p* = 0.008, partial η^2^ = 0.031). Specifically, the masculine group had higher RA scores than the feminine group (t = 2.57, *p* = 0.011). Although the sex-by-gender role interaction was not significant (F_1,230_ = 1.86, *p* = 0.174, partial η^2^ = 0.008), we performed the simple-effects analyses. The results indicated that RA did not differ significantly by sex in either the masculine group (*p* = 0.087) or the feminine group (*p* = 0.842), as shown in Figure 2B.

### 3.3. Additional Findings on RA

The above results demonstrated whether the effect of sex on RA varies by gender role orientation. Subsequently, we examined whether the effect of gender role orientation on RA varies by sex. Contrasting the two findings allows us to determine which factor exerts a greater influence on RA. As shown in Figure 2B, there was no significant difference in RA between masculine males and feminine males (*p* = 0.362), but RA was significantly higher in masculine females than in feminine females (*p* = 0.003).

### 3.4. Sex Differences in PA Across Different Gender Role Orientations

**Figure 3:**
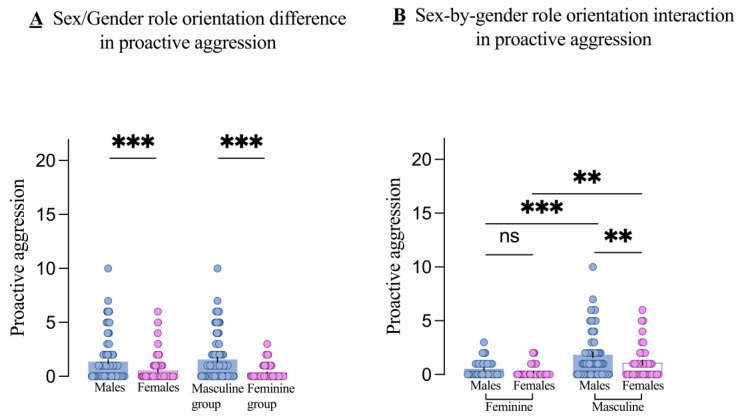
Main effects and interactions on proactive aggression. The main effect of sex (males and females) and gender role orientation (masculine and feminine groups) in proactive aggression (**A**) and the sex-by-gender role orientation interaction on proactive aggression (**B**). Each dot is a participant. All error bars reflect the standard error of the mean. ns = not significant. ** *p* < 0.01; *** *p* < 0.001.

Regarding PA, both the main effects of sex (*F*_1, 230_ = 7.12, *p* = 0.008, partial η^2^ = 0.030) and gender role orientation (*F*_1,230_ = 30.35, *p* < 0.001, partial η^2^ = 0.117) were significant. Post hoc tests revealed that males had higher PA scores than females (*t* = 4.28, *p* < 0.001), and the masculine group had higher PA scores than the feminine group (*t* = 6.49, *p* < 0.001), as shown in Figure 3A. Although the sex-by-gender role interaction was not significant (*F*_1,230_ = 1.29, *p* = 0.257, partial η^2^ = 0.006), simple-effects analysis showed a significant sex difference, with males having higher PA scores than females in the masculine group (*p* = 0.008), but no significant difference in the feminine group (*p* = 0.277), as shown in Figure 3B.

### 3.5. Additional Findings on PA

Similarly, we examined whether the effect of gender role orientation on PA varies by sex. Results showed that PA scores were higher in masculine males compared to feminine males (*p* < 0.001) and higher in masculine females compared to feminine females (*p* = 0.002), as shown in Figure 3B.

### 3.6. Sex and Gender Role Orientation Effects on GMV

There was a significant main effect of sex on GMV across multiple regions, including the cerebellum, temporal lobe, parietal lobe, occipital lobe, frontal lobe, limbic system, and basal ganglia (Table 4, Part C of Supplementary Material S1 and S2). In clusters 1–9, males exhibited larger GMV than females, while in cluster 10, the pattern was reversed.

There was also a significant main effect of gender role orientation on GMV in the left middle temporal gyrus (MTG, x = −54, y = −22.5, z = −10.5, *T* = 4.13). The masculine group had significantly larger left MTG GMV than the feminine group (0.66 vs. 0.60, *t* = 5.37, *p* < 0.001, Table 5).

**Figure 4:**
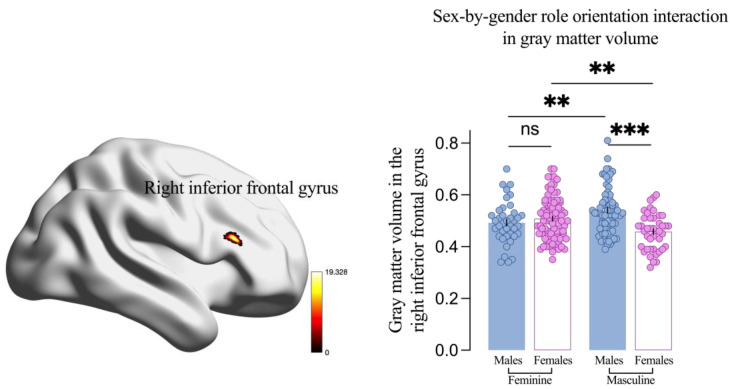
Interaction between sex and gender role orientation on gray matter volume in the right inferior frontal gyrus. Results are threshold at the cluster *p* < 0.05 and voxel-level *p* < 0.001 by Gaussian random field correction. Each dot is a participant. All error bars reflect the standard error of the mean. ns = not significant. ** *p* < 0.01; *** *p* < 0.001.

More importantly, a significant sex-by-gender role orientation interaction on GMV in the right inferior frontal gyrus (IFG, x = 48, y = 25.5, z = 19.5, *F* = 19.33) was found. Specifically, GMV in the right IFG was larger in males than females within the masculine group (0.54 vs. 0.46, *p* = 0.008) but not within the feminine group (0.49 vs. 0.51, *p* = 0.374, Figure 4). Additionally, GMV in the right IFG was larger in masculine males compared to feminine males (0.54 vs. 0.49, *p* = 0.005) but smaller in masculine females compared to feminine females (0.46 vs. 0.51, *p* = 0.002, Figure 4).

### 3.7. Relationship Between Aggression and Brain GMV

Firstly, considering the main effects of sex and gender role orientation on aggression and brain GMV, we explored the correlation between aggression and GMV in regions that showed a significant main effect (Table 4 and Table 5). Results showed that PA was positively related to GMV in several regions where a significant main effect of sex was found, including clusters 1 and 2, and negatively related to cluster 10. Additionally, PA was positively correlated with GMV in the left MTG; the region showed a significant main effect of gender role orientation.

**Figure 5:**
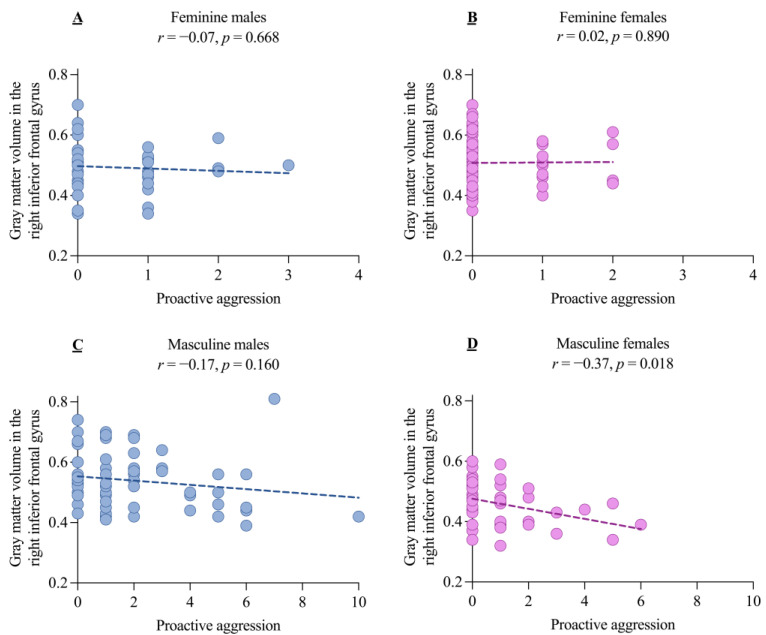
The correlation between gray matter volume in the right inferior frontal gyrus and proactive aggression for the four samples orthogonalized by sex and gender role orientation. The correlations between the gray matter volume in the right inferior frontal gyrus and proactive aggression in feminine males (**A**), feminine females (**B**), masculine males (**C**), and masculine females (**D**).

Secondly, given the aforementioned results (i.e., higher PA and right IFG GMV in males compared to females within the masculine group), we investigated the correlation between aggression and the right IFG GMV across the four groups. A significant negative correlation was found between PA and the right IFG GMV in the masculine females, but no significant correlations were found in the masculine males and the other two groups (all *p* > 0.05, Figure 5).

Thirdly, no significant relationship was found between RA and GMV in regions that showed a significant main effect (Table 5) or interaction effect (feminine males: *r* = −0.13, *p* = 0.450; feminine females: *r* = 0.10, *p* = 0.377; masculine males: *r* = −0.11, *p* = 0.391; masculine females: *r* = −0.12, *p* = 0.458).

## 4. Discussion

This study examined the interaction between sex and gender role orientation on aggression and brain GMV to determine whether sex differences in aggression are uniquely explained by sex, gender role orientation, or their interaction and to explore the neuroanatomical characteristics underlying these sex differences. After dissociating the effects of sex and gender role orientation on RA, PA, and brain GMV, this study confirmed that sex differences in PA (males > females) and right IFG GMV (males > females) were present within the masculine group, but not within the feminine group. Moreover, PA was negatively associated with right IFG GMV in masculine females compared to masculine males.

### 4.1. Interaction Analyses on Aggression

First, we found a main effect of sex on PA. Previous research has consistently demonstrated higher PA in males than females [43,44]. According to social learning theory, boys are often taught to be strong and physically protective, while girls are taught to avoid danger through the influence of parents, peers, and social media. This socialization process can contribute to sex differences in PA [45].

Second, the masculine group reported higher RA and PA than the feminine group. This result aligns with previous findings that masculine participants engaged in more cyber aggression than feminine participants [46]. It is important to note that tendencies for aggression are heavily influenced by individuals’ social experiences [47]. Social role theory proposes that masculine roles typically involve higher-status social positions, whereas feminine roles tend to emphasize caregiving and nurturing. These distinct roles can reinforce or diminish attitudes toward power and competition, which may, in turn, influence aggression [48].

Third, after dissociating the effects of sex and gender role orientation on RA and PA, this study indicated that feminine males were more similar to feminine females than masculine males were to masculine females on PA. Prior research suggests that masculine boys are more prone to relational aggression than masculine girls [46], and this type of aggression is more related to PA than RA [49], this is consistent with our findings. These phenomena can be attributed to a combination of ‘traits’ and ‘opportunities’. Compared to masculine females, masculine males are more likely to undergo socialization processes that emphasize social dominance and aggression due to their biological sex, providing them with more opportunities to display aggressive tendencies [46]. In contrast, although feminine males experience similar socialization processes that emphasize aggression, their inherent feminine traits may cause them to be more cautious in seizing opportunities to display aggression.

Meanwhile, the key findings were that the impact of sex on aggression within the same gender role orientation is relatively minimal (only manifested in PA: masculine males > masculine females). Conversely, the impact of gender role orientation on aggression within the same sex is relatively greater (RA and PA: masculine females > feminine females; PA: masculine males > feminine males). These findings demonstrate that gender role orientation has a more significant effect on aggression than sex, particularly regarding PA.

### 4.2. GMV in Different Brain Regions and Aggression

The assumption that “the larger the volume, the better the function” is not necessarily correct, and it is misleading to interpret GMV data in isolation [50]. Therefore, this section discusses the relationship between GMVs and aggression.

First, significant associations were found between PA and several brain regions, with a significant main effect of sex or gender role orientation. The larger clusters 1 and 2 in males or the larger right MTG GMV in the masculine group, the higher the PA. Conversely, the larger cluster 10 in females, the lower the PA. A study of 5216 participants from the UK Biobank showed that males had larger GMV across all cortical subregions [51], consistent with our findings. Clusters 1 and 2 also involve many cortical subregions, including the temporal lobe, parietal lobe, and occipital lobe, in contrast to prior studies on children aged 7–17, which found a positive correlation between temporal lobe GMV and femininity [24], our study of young adults (17–22) found that the feminine group had lower left MTG GMV. We speculate that this reflects earlier cortical pruning in feminine individuals [24,26].

Second, after dissociating the effects of sex and gender role orientation on GMV, a significant interaction on the frontal lobe, specifically the right IFG, was found. This result aligns with previous findings [26], and our data also suggest that the right IFG is related to aggression. On the one hand, sex differences in right IFG GMV were observed in the masculine group (males > females) but not in the feminine group. The IFG is crucial for cognitive control [52]. Compared to masculine males, masculine females may experience greater inhibition in PA-related behaviors due to social expectations that discourage highly instrumental and competitive behaviors in favor of expressivity [21]. Research shows that females who deviate from gender roles are more likely to be assaulted [53]. Female self-esteem is more influenced by external evaluation, particularly negative ones [54]. Therefore, successful cognitive control in masculine females may aim to reduce PA, and the negative correlation between right IFG GMV and PA supports the idea.

On the other hand, although there was no significant correlation between right IFG GMV and PA in masculine males, they exhibited both higher right IFG GMV and PA. This provides indirect evidence for the emerging view of aggression as successful self-control in (masculine) males [55].

Interestingly, we found that masculine females scored significantly higher in RA compared to feminine females, and masculine females had significantly lower GMV in the right IFG compared to feminine females. The IFG is closely associated with response inhibition [56], and the reduction in IFG GMV may indicate that masculine females have a deficit in inhibiting inappropriate responses, leading to their elevated levels of RA. Overall, the increased levels of RA and PA in masculine females may both be attributed to their failure in self-control.

### 4.3. Limitations

First, this study is the exclusive reliance on GMV as the neuroimaging measure, while GMV reveals structural differences, it does not capture functional brain dynamics. Future research should integrate functional techniques, such as fMRI, to provide a more comprehensive understanding of the relationship between brain structure, function, and gender roles. Additionally, PA was relatively low across all subjects, likely due to our sample of college students in higher education. Studying groups with higher levels of aggression would help expand these findings. Third, our study was cross-sectional and non-interventional, so causation cannot be inferred. Longitudinal studies, functional MRI, or non-invasive brain stimulation may provide further insight into these relationships. Finally, masculinity and femininity are heavily influenced by culture, which may limit the generalizability of our findings to other populations and cultural contexts.

## 5. Conclusions

The differential effects of sex and gender role orientation on RA and PA highlight the greater influence of gender role orientation compared to sex. For RA, being male is not a marker; instead, masculine traits serve as a marker of RA, specifically in females. The elevated RA observed in masculine females, compared to feminine females, may be linked to impaired response inhibition, as indicated by reduced GMV in the IFG. For PA, being male is a marker only within the masculine group, whereas masculine traits emerge as a more robust marker of PA across both males and females. In masculine females, elevated PA is associated with reduced IFG GMV, reflecting impaired cognitive control. In contrast, in masculine males, heightened PA corresponds to effective cognitive control, as evidenced by higher IFG GMV.

## Figures and Tables

**Table 1:** Demographic characteristics of the masculine and feminine groups.

	Males		Females	
	Age (*M* ± *SD*)	*n*	Age (*M* ± *SD*)	*n*
Masculine group	19.16 ± 0.94	69	18.99 ± 0.81	42
Feminine group	19.17 ± 0.82	39	19.08 ± 0.96	84

Note. *M* = mean; *SD* = standard deviation.

**Table 2:** Gender role orientation measures for the four groups orthogonalized by sex and gender role orientation.

		Males	Females	*t*
Masculine group	Masculinity	4.93 ± 0.34	4.98 ± 0.39	−0.82
	Femininity	4.40 ± 0.31	4.31 ± 0.31	1.31
	*t*	9.31 ***	9.64 ***	
Feminine group	Masculinity	3.93 ± 0.42	3.98 ± 0.39	−0.64
	Femininity	5.07 ± 0.30	5.22 ± 0.37	−2.17 *
	*t*	−12.18 ***	−22.35 ***	

Note. * *p* < 0.05; *** *p* < 0.001. The masculinity and femininity scores are mean ± standard deviation.

**Table 3:** Means and standard deviations on RA and PA in the four groups.

	Masculine Males	Masculine Females	Feminine Males	Feminine Females
RA	7.03 ± 4.34	8.36 ± 3.69	6.31 ± 3.74	6.15 ± 3.81
PA	1.84 ± 2.18	1.10 ± 1.54	0.54 ± 0.76	0.24 ± 0.53

Note. RA = reactive aggression; PA = proactive aggression.

**Table 4:** Main effect of sex on clusters 1–10 and the relationship between the significant regions and the two subtypes of aggression.

	Males	Females	*t*	*p*	*r* (RA)	*p* (RA)	*r* (PA)	*p* (PA)
Cluster 1	0.67 ± 0.05	0.60 ± 0.04	12.83	<0.001	−0.057	0.384	0.137	0.036
Cluster 2	0.65 ± 0.05	0.58 ± 0.04	12.01	<0.001	−0.022	0.734	0.200	0.002
Cluster 3	0.60 ± 0.07	0.53 ± 0.06	9.08	<0.001	−0.065	0.319	0.028	0.667
Cluster 4	0.55 ± 0.07	0.49 ± 0.06	7.89	<0.001	−0.024	0.714	0.101	0.124
Cluster 5	0.52 ± 0.05	0.46 ± 0.06	8.97	<0.001	0.018	0.780	0.100	0.129
Cluster 6	0.58 ± 0.07	0.51 ± 0.06	8.61	<0.001	0.001	0.985	0.099	0.130
Cluster 7	0.56 ± 0.08	0.49 ± 0.07	7.36	<0.001	−0.097	0.141	0.027	0.676
Cluster 8	0.66 ± 0.06	0.61 ± 0.06	7.39	<0.001	−0.044	0.506	0.116	0.076
Cluster 9	0.62 ± 0.08	0.54 ± 0.06	7.93	<0.001	−0.051	0.439	0.127	0.052
Cluster 10	0.58 ± 0.07	0.61 ± 0.07	−2.97	0.003	0.008	0.903	−0.13	0.047

Note. Part C of the Supplementary Material S1 provides further details. RA = Reactive aggression; PA = Proactive aggression. The gray shading represents the correlation analysis. The sizes of the clusters are mean ± standard deviation. Cluster 1 refers to an extensive brain region that includes the temporal lobe, parietal lobe, occipital lobe, cerebellum, limbic system, and basal ganglia. Cluster 2 refers to another extensive brain region that encompasses the temporal lobe, limbic system, and basal ganglia. Cluster 3 = Right middle frontal gyrus. Cluster 4 = Right supplementary motor area. Cluster 5 = Left superior frontal gyrus, medial. Cluster 6 = Right middle frontal gyrus. Cluster 7 = Right supramarginal gyrus. Cluster 8 = Vermis_8. Cluster 9 = Angular gyrus. Cluster 10 = Right ventral lateral gyrus.

**Table 5:** Main effect of gender role orientation on left middle temporal and the relationship between the regional gray matter volume and the two subtypes of aggression.

	Masculine Group	Feminine Group	*t*	*p*	*r* (RA)	*p* (RA)	*r* (PA)	*p* (PA)
Left middle temporal gyrus	0.66 ± 0.09	0.60 ± 0.08	5.37	<0.001	−0.015	0.817	0.142	0.029

Note. The gray shading represents the correlation analysis. The sizes of the clusters are mean ± standard deviation.

## Data Availability

Where reasonable, data are available from the corresponding author. The data are not publicly available to protect participants’ privacy.

## Supplementary Materials

The following supporting information can be downloaded at https://www.mdpi.com/article/10.3390/brainsci14121176/s1. Part A of S1: Measurement of sex, gender role orientation, RA, and PA [9,46,21,57,58,59,60,61,62,63]; Part B of S1: MRI data acquisition and processing [64,65,66,67]; Figure S1: Brain regions involved in cluster 1; Figure S2: Brain regions involved in cluster 2; Table S1: Significant main effect of sex on gray matter volume; Excel S2: Visualizable tables of clusters 1 and 2.
