# Supplementary material for: Is Being Male a Marker of Aggression? Evidence for the Decoupling of Sex and Gender Role Orientation
Source: Brain Sci. 2024 Nov 25;14(12):1176. doi: 10.3390/brainsci14121176 (PMC11674439; doi:10.3390/brainsci14121176)
Supplement: Supplementary file 1 [file brainsci-14-01176-s001.zip › Supplementary Material S1.pdf]

Part A of Supplementary Material S1

Measurement of sex, gender role orientation, RA, and PA

Sex

This study defines sex as an individual's sex assignment at birth [1]. We collected each subject's current designated sex as indicated on their resident identity card. None of the participants reported undergoing sex reassignment surgery, so the gender on their current identification card represents the sex assigned at birth. It should be noted that China only identifies two sexes assigned at birth (male and female).

Gender role orientation

Masculine and feminine traits were assessed using the Bem Sex Role Inventory [2]. It comprises 20 items related to femininity (e.g., cheerful and understanding) and 20 related to masculinity (e.g., independent and forceful). Participants were asked to rate their identification with culturally prescribed masculinity and femininity traits on a 7-point scale ranging from 1 (*never or almost never true*) to 7 (*always or almost always true*). Elevated scores on this inventory signify a higher degree of masculinity or femininity. This scale has been validated and widely used to measure masculinity and femininity in many countries [3], including China [4,5].

Although the Bem Sex Role Inventory is considered old, it focuses on assessing traditional aspects of masculinity and femininity that are more closely associated with aggression [6,7]. Furthermore, albeit with evolving connotations of masculinity and femininity as society progresses, recent studies have suggested that women's and men's perceptions of gender roles are similar to those of previous eras [8]. In our study, Cronbach's alpha of the femininity and masculinity subscale was 0.82 and 0.87, respectively.

RA and PA

The Reactive and Proactive Aggression Questionnaire (Raine et al., 2006) was used to measure RA and PA [9]. This instrument consists of 23 items rated on a 3-point scale from 0 (*never*) to 2 (*often*). RA was assessed by 11 items (e.g., "Gotten angry when frustrated") and PA by12 items (e.g., "Yelled at others so they would do things for you"). The scale has been validated and widely used for measuring RA and PA [10]. In the study, the RA and PA subscales had a Cronbach's alpha of 0.81 and 0.83, respectively.

Part B of Supplementary Material S1

MRI data acquisition and processing

All images were first displayed in SPM12 to determine whether there were artifacts or gross anatomical abnormalities and set to the anterior-posterior commissure line for better registration. Second, the anatomical images were segmented into white matter (WM), gray matter (GM), and cerebrospinal fluid (CSF) using the new segmentation routine in SPM12 [11]. Third, we conducted registration, normalization, and modulation using the Diffeomorphic Anatomical Registration Through Exponential Lie Algebra method (DARTEL) [12], which has been extensively used in previous VBM studies [13,14]. Specifically, the GM images were resampled to 1.5 mm × 1.5 mm × 1.5 mm, normalized to the Montreal Neurological Institute (MNI) space, and smoothed with a 6 mm Full Width at Half Maximum Gaussian (FWHM) kernel. Finally, to ensure that local GM volumes were conserved, the image intensity of each voxel was modulated.

Part C of Supplementary Material S1

Table S1

Significant main effect of sex on gray matter volume

	Anatomical region (Peak label)	Cluster size	x	y	z	*T*
	Males > Females					
1	Right lingual gyrus	33512	13.5	-93	-15	10.49
2	Left paraHippocampal gyrus	6278	-18	-10.5	-30	8.20
3	Right middle frontal gyrus	2617	36	52.5	18	5.07
4	Right supplementary motor area	863	10.5	6	60	4.71
5	Left superior frontal gyrus, medial	834	-6	52.5	31.5	4.46
6	Right middle frontal gyrus	685	40.5	16.5	48	4.78
7	Right supramarginal gyrus	450	67.5	-39	22.5	4.34
8	Vermis_8	333	0	-75	-39	5.31
9	Right angular gyrus	293	57	-64.5	25.5	4.35
	Females > Males					
10	Right ventral lateral gyrus	440	10.5	-12	6	-5.63

Note. The results were thresholded at $p < 0.05$ for clusters and $p < 0.001$ for voxels using Gaussian random field correction (Two-tailed). The provided Supplementary Material 2 enables sorting by hemisphere, region, and cluster size, allowing easy access to the needed information.

Figure S1

Brain regions involved in cluster 1

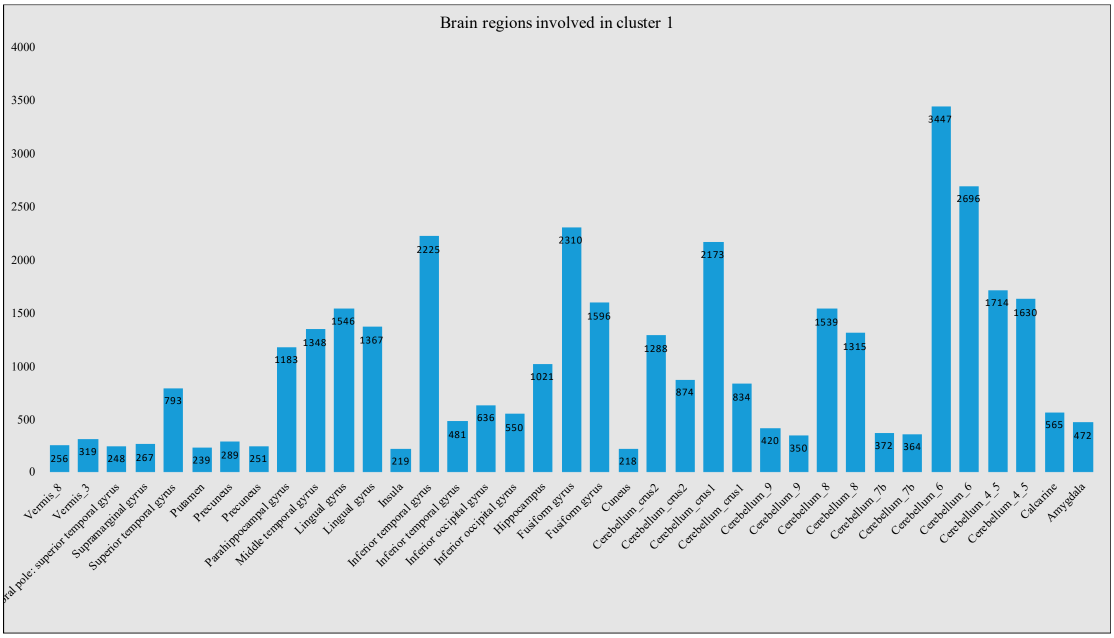

Note. The cluster 1 including the cerebellum (i.e., vermis_3, vermis_8, cerebellum_crus1, cerebellum_crus2, cerebellum_4_5, cerebellum_6, cerebellum_7b, cerebellum_8, and cerebellum_9), temporal lobe regions (i.e., superior temporal gyrus, temporal pole, middle temporal gyrus, inferior temporal gyrus, and fusiform gyrus), the parietal lobe regions (i.e., supramarginal gyrus and precuneus), the limbic system (i.e., parahippocampal gyrus, hippocampus, insula, and amygdala), the basal ganglia (i.e., putamen), the occipital lobe (i.e., lingual gyrus, cuneus, inferior occipital gyrus, and calcarine)

Figure S2

Brain regions involved in cluster 2

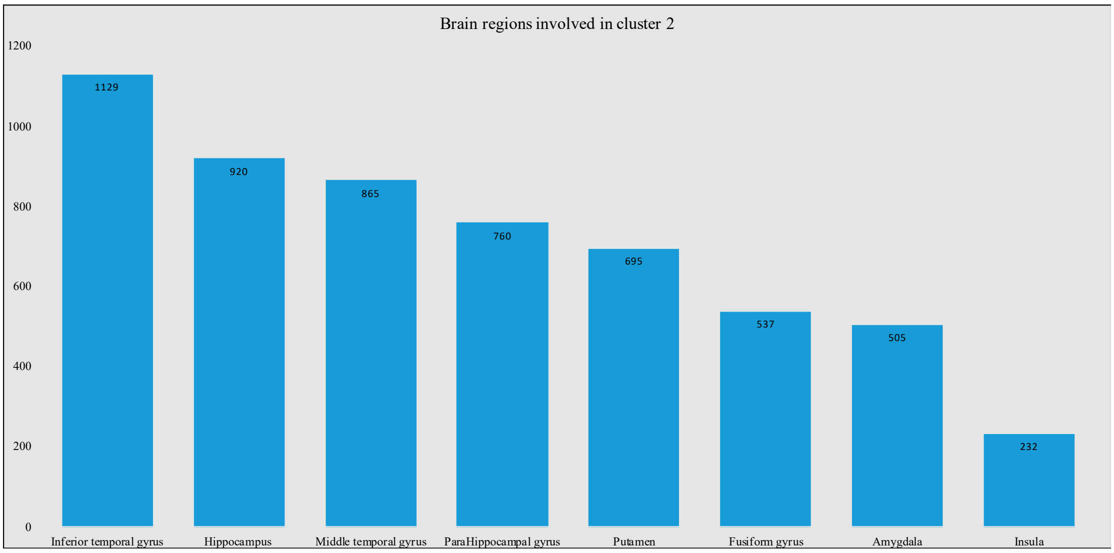

Note. The cluster 2 including the temporal lobe (i.e., inferior temporal gyrus, middle temporal gyrus, and fusiform gyrus), the limbic system (i.e., hippocampus, parahippocampal gyrus, insula, and amygdala), and the basal ganglia (i.e., putamen and insula).

References

1. Tate, C.C.; Ledbetter, J.N.; Youssef, C.P. A two-question method for assessing gender categories in the social and medical sciences. *Journal of sex research* **2013**, *50*, 767-776, doi:10.1080/00224499.2012.690110.
2. Bem, S.L. The measurement of psychological androgyny. *Journal of consulting and clinical psychology* **1974**, *42*, 155, doi:10.1037/h0036215.
3. Hsu, N.; Badura, K.L.; Newman, D.A.; Speach, M.E.P. Gender,"masculinity," and "femininity": A meta-analytic review of gender differences in agency and communion. *Psychological Bulletin* **2021**, *147*, 987, doi:10.1037/bul0000343.
4. Zhang, J.; Norvilitis, J.M.; Jin, S. Measuring Gender Orientation with the Bem Sex Role Inventory in Chinese Culture. *Sex Roles* **2001**, *44*, 237-251, doi:10.1023/A:1010911305338.
5. Lo, I.P.Y.; Kim, Y.K.; Small, E.; Chan, C.H.Y. The Gendered Self of Chinese Lesbians: Self-Esteem as a Mediator Between Gender Roles and Depression. *Archives of Sexual Behavior* **2019**, *48*, 1543-1554, doi:10.1007/s10508-019-1402-0.
6. Malonda, E.; Llorca, A.; Zarco Alpuente, A.; Samper, P.; Mestre, V. Linking Traditional Masculinity, Aggression, and Violence. 2022; 10.1007/978-3-030-98711-4_35-1pp. 1-26.
7. Wright, M.F. The role of technologies, behaviors, gender, and gender stereotype traits in adolescents' cyber aggression. *Journal of interpersonal violence* **2020**, *35*, 1719-1738, doi:10.1177/0886260517696858.
8. Haines, E.L.; Deaux, K.; Lofaro, N. The times they are a-changing… or are they not? A comparison of gender stereotypes, 1983–2014. *Psychology of Women Quarterly* **2016**, *40*, 353-363, doi:10.1177/0361684316634081.
9. Raine, A.; Dodge, K.; Loeber, R.; Gatzke-Kopp, L.; Lynam, D.; Reynolds, C.; Stouthamer-Loeber, M.; Liu, J. The reactive–proactive aggression questionnaire: Differential correlates of reactive and proactive aggression in adolescent boys. *Aggressive Behavior: Official Journal of the International Society for Research on Aggression* **2006**, *32*, 159-171, doi:10.1002/ab.20115.
10. Fite, P.J.; Rubens, S.L.; Preddy, T.M.; Raine, A.; Pardini, D.A. Reactive/proactive aggression and the development of internalizing problems in males: The moderating effect of parent and peer relationships. *Aggressive Behavior* **2014**, *40*, 69-78, doi:https://doi.org/10.1002/ab.21498.
11. Ashburner, J.; Friston, K.J. Unified segmentation. *Neuroimage* **2005**, *26*, 839-851, doi:10.1016/j.neuroimage.2005.02.018.
12. Ashburner, J. A fast diffeomorphic image registration algorithm. *Neuroimage* **2007**, *38*, 95-113, doi:10.1016/j.neuroimage.2007.07.007.
13. Vega, D.; Ripolles, P.; Soto, A.; Torrubia, R.; Ribas, J.; Monreal, J.A.; Pascual, J.C.; Salvador, R.; Pomarol-Clotet, E.; Rodriguez-Fornells, A., et al. Orbitofrontal overactivation in reward processing in borderline personality disorder: the role of non-suicidal self-injury. *Brain Imaging Behav.* **2018**, *12*, 217-228, doi:10.1007/s11682-017-9687-x.
14. Kobayashi, A.; Yokota, S.; Takeuchi, H.; Asano, K.; Asano, M.; Sassa, Y.; Taki, Y.; Kawashima, R. Increased grey matter volume of the right superior temporal gyrus in healthy children with autistic cognitive style: A VBM study. *Brain Cogn.* **2020**, *139*,

105514, doi:10.1016/j.bandc.2019.105514.
